# Strength Development of High-Strength Ductile Concrete Incorporating Metakaolin and PVA Fibers

**DOI:** 10.1155/2014/387259

**Published:** 2014-02-23

**Authors:** Muhammad Fadhil Nuruddin, Sadaqat Ullah Khan, Nasir Shafiq, Tehmina Ayub

**Affiliations:** Civil Engineering Department, Universiti Teknologi PETRONAS, Block 13, Level III, 31750 Tronoh, Perak, Malaysia

## Abstract

The mechanical properties of high-strength ductile concrete (HSDC) have been investigated using Metakaolin (MK) as the cement replacing material and PVA fibers. Total twenty-seven (27) mixes of concrete have been examined with varying content of MK and PVA fibers. It has been found that the coarser type PVA fibers provide strengths competitive to control or higher than control. Concrete with coarser type PVA fibers has also refined microstructure, but the microstructure has been undergone with the increase in aspect ratio of fibers. The microstructure of concrete with MK has also more refined and packing of material is much better with MK. PVA fibers not only give higher stiffness but also showed the deflection hardening response. Toughness Index of HSDC reflects the improvement in flexural toughness over the plain concrete and the maximum toughness indices have been observed with 10% MK and 2% volume fraction of PVA fibers.

## 1. Introduction

Since the industrial revolution, large economic and industrial growth caused a massive increase in cement and steel usage which are main building construction materials. The requirement of cement and steel has been amplified about 84% and 64% during the last decade [1, 2]; especially the race of tallest building construction augmented the consumption of cement and steel greatly. Also, the cement and steel producing industries contribute about 5% and 7% of global CO_2_ emissions [3, 4] and today concrete industry is the largest consumer of natural resources such as water, sand, gravel, and crushed rock [5]. Indeed, the volume of depletion of natural resources and production of CO_2_ is very large. Each year about 14% and 20% of global industrial energy are consumed by cement and steel manufacturing industries [6]; therefore, for sustainable and environmentally viable development, large production of cement and steel is undesirable and gradual reduction in the use of cement and steel is needed. Till date, several researchers investigated the supplementary materials for cement and steel, but cement and steel cannot be completely replaced with any other supplementary material. Cement can only be partially supplemented by mineral admixtures such as fly ash, silica fume, ground granulated blast furnace slag, rice husk ash, and Metakaolin (MK) and use of steel can be partially reduced by introducing ductility in concrete.

Metakaolin (MK) during the last two decades gets recognition as mineral admixture. The general chemical and physical properties of MK with other mineral admixtures and ordinary Portland cement (OPC) are shown in Table 1. MK possesses substantial content of silica and alumina in comparison with cement and other mineral admixtures showing the capability to produce both strengthening gel, that is, calcium silicate hydrate (CSH) and calcium aluminates hydrate (CAH) by reacting with the primary hydrate of cement. The maximum compressive strength achieved by using MK in concrete is 134 MPa [7]. The early strength gained is higher with the addition of MK in comparison with fly ash and silica fume [8]. Also increase in the tensile and bending strength of concrete and mortar with 10 to 15% MK is 32% and 38%, respectively, and it is better than silica fume [9, 10]. In 2002, effect of metakaolin and silica fume on compressive strength, chloride diffusivity, and shrinkage has been studied [11], and it has been found that metakaolin and silica fume are comparable and give higher early strength, reduce chloride diffusivity, and reduce drying shrinkage. In 2005, these results have been reconfirmed and it has also been reported that both metakaolin and silica fume give low porosity and smaller pore size and results improved microstructure of concrete [12].

Low tensile strength of concrete is due to the propagation of single internal crack. If the crack restrained locally by extending into another matrix adjacent to it, the initiation of crack is retarded and higher tensile strength of concrete is achieved [13]. This restrained can be achieved by adding small length fibers to concrete. In addition to increasing the tensile strength, addition of fibers enhances fatigue resistance [14], energy absorption, toughness, ductility, and durability [15]. Properties of few reinforcing fibers that have been used in cementitious composites are shown in Table 2. Based on modulus of elasticity, fibers are further classified into two basic categories, namely, hard intrusion and soft intrusion. Those fibers having a high modulus of elasticity than cement mortar are called hard intrusion and those with lower modulus of elasticity than cement mortar are called soft intrusion [16]. Steel, carbon, and glass are the hard intrusion fibers, and polypropylene and vegetable fibers are the soft intrusion fibers. fibers having a low modulus of elasticity are unlikely to improve strength but improve the resistance against impact and shock due to elongation ability. However, fibers having a higher modulus of elasticity make concrete strong and stiff [16].

Polyvinyl alcohol (PVA), an organic fiber, was explored 50 years ago by Japanese and has been used in cement applications since the 1980s due to suitable characteristics as reinforcing materials for cementitious composites. PVA fibers have tensile strength and young's modulus higher than other organic fibers. Because of higher modulus of elasticity, PVA fibers perform better for cracking control. Finer type PVA fiber has been used for fiber cement roofing replacing asbestos. Coarse PVA has been used in tunnel lining, industrial floor, and roadbed overlays [17]. The most important characteristics of PVA fiber is the strong bond with cement matrix, higher modulus of elasticity, and bond strength of PVA. These add more flexibility and tensile strength in concrete [18]. PVA fibers like steel and glass fibers are most promising in terms of tensile strength, young's modulus and fiber elongation as shown in Table 2.

Using mineral admixtures, along with superplasticizer with low water content, very dense high-strength concrete (HSC) has been obtained [19], but due to brittle nature, it requires steel reinforcement in most of engineering applications [20]. Ultrahigh performance fiber reinforced concrete (UHPFRC) addresses the issue of the brittleness of HSC by utilizing steel fiber in high-strength concrete to induce ductility in concrete. UHPFRC is a high-strength, durable, and ductile concrete, but there are few concerns such as high cost [21], fire resistance [22], and high thermal conductivity [23]. In UHPFRC, to avoid improper packing of material due to fibers, fibers are used with/without the coarse aggregates. Absence of coarse aggregate requires increase in fine aggregate. Also, higher content of cement is required due to the higher content of fine aggregates. Further, the modulus of elasticity of UHPFRC is decreased due to absence of coarse aggregates.

The use of steel fibers in fiber reinforced concrete (FRC) and in UHPFRC has been widely studied; however limited research has been carried out on the effect of other reinforcing fibers in concrete. Moreover, High-performance fiber reinforced concrete has been studied with silica fume and metakaolin with the steel fiber, and it has been found that metakaolin has better performance with steel fibers [24]. Cementitious materials such as fly ash, silica fume, or ground granulated blast furnace slag with steel, polypropylene, or PVA fibers have been studied widely [25–27], but MK with PVA fibers has not been studied for developing of high-strength ductile concrete (HSDC).

The basic focus of the current study is to use MK and PVA fibers together in order to lessen the use of cement and steel without compromising the performance. The performance criteria are the mechanical properties of concrete with MK and PVA fibers. Cube compressive strength, splitting tensile strength, and flexural strength of concrete have been observed to check the effect of MK and PVA fibers. Also, the microstructure of HSDC has been examined through field emission scanning electron microscope and results of microstructure and compressive strength has been compared.

## 2. Experimental Program

Twenty-seven (27) mixes of concrete have been prepared; three (03) out of twenty-seven (27) are control mixes without PVA fibers. Nomenclature and detail of mix proportion of concrete have been mentioned in Table 3. Three specimens for all concrete mixes have been prepared to determine the average of each mechanical property, that is, compressive strength, splitting tensile strength, and flexural strength. The effect of % volume fraction of PVA fibers, the aspect ratio of PVA fibers and cement replacement with MK on the compressive strength, splitting tensile strength, and flexural strength of concrete at 7 days, 28 days, 56 days, and 90 days has been investigated.

### 2.1. Materials

Ordinary Portland cement (OPC) has been used. Sand passing through 3.125 mm (1/8^”^) opening sieve and retained on sieve no. 200 has been used as fine aggregates in all concrete mix. 20 mm (3/4^”^) opening sieve passing and 10 mm (3/8^”^) opening sieve passing have been used as coarse aggregates. Polyvinyl alcohol (PVA) fibers have been selected due to their reinforcing properties. Length-diameter ratio is one of the parameters of the current study, which has been varied from 45 to 120, and fiber volume fraction has been varied from 1% to 2% by volume of concrete. PVA fiber of aspect ratio 45 is coarser (Diameter 0.667 mm) while PVA fibers of aspect ratio higher than 45 are finer (Diameter 0.2 mm). Locally available kaolin has been calcined to form MK and it has been used as mineral admixture. The physical and chemical properties of MK have been mentioned in Table 1. MK has been used 5 to 10% by weight of cement in concrete mixes as cement replacing material. Superplasticizer of Type F or G of ASTM C 494 with a dosage 0.25 to 1.25% by weight of binder with a variation of 0.25% has been added in concrete mixes to achieve the slump of 50 ± 10 mm in all concrete mix and water binder ratio has been taken as 0.4 in all concrete mixes.

### 2.2. Details of Experiments

Mechanical properties (compressive strength, splitting tensile strength, and flexural strength) of HSDC have been tested based on BS standards. Prior to testing, all concrete mixes have been tested for slump test to set a slump of 50 ± 10 mm. Size of all specimens has been set up as per BS standard to determine mechanical properties. Specimens have been cast using steel moulds to avoid any attack of cement. Slump test has been performed according to BS 1881 to set up the optimum content of superplasticizer for the workability of concrete. Apparatus, operation, and standards have been adapted to comply with BS 1881: Part 102 standards.

#### 2.2.1. Compressive Strength

All concrete mixes have been prepared to determine the mechanical properties of concrete. Specimens for determining compressive strength of concrete have been cast according to BS 1881: Part 108. Specimen of 100 × 100 × 100 mm cube has been used for testing of compressive strength.

#### 2.2.2. Splitting Tensile Strength

Specimens for determining the splitting tensile strength of concrete have been cast as per BS 1881: Part 110. Cylinder of 150 mm diameter and 150 mm height has been used for testing of splitting tensile strength.

#### 2.2.3. Flexural Strength

Specimens for determining the flexural strength of concrete have been cast as per BS 1881: Part 109. Beam of 100 × 100 × 500 mm has been used for testing of flexural strength.

## 3. Results and Discussion

**Figure 1:**
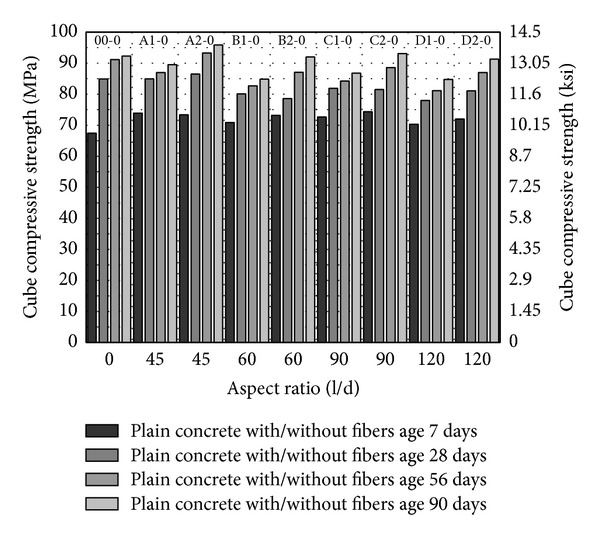
Cube compressive strength of HSDC without metakaolin.

**Figure 2:**
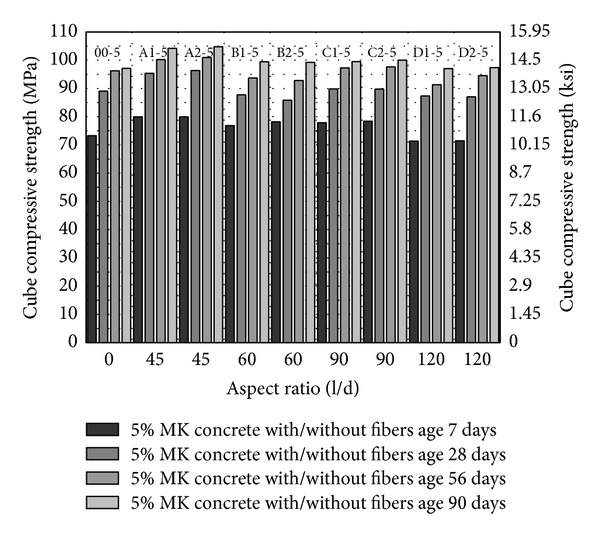
Cube compressive strength of HSDC with 5% metakaolin.

**Figure 3:**
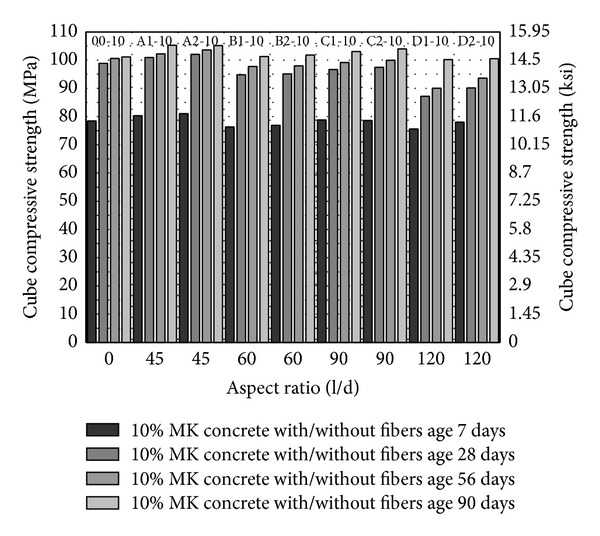
Cube compressive strength of HSDC with 10% metakaolin.

From Figure 1 to Figure 3, cube compressive strength of concrete has been plotted. Compressive strength has been plotted against aspect ratio while volume fraction and age of specimen have been shown in the series. Control mixes have been plotted by putting aspect ratio zero. The most obvious trend in these plots is the increase in compressive strength with the age and with increase in volume fraction of fibers from 1% to 2%. The highest cube compressive strength has been observed with 2% volume fraction of fibers having aspect ratio 45 and 5–10% MK (A2-5, A2-10) as shown in Figures 2 and 3.

In Figure 1, compressive strength of concrete without MK has been shown. Compressive strength of concrete with the fibers having aspect ratio 45 is higher as shown in Figure 1; however, it is comparable with control. With the increase in aspect ratio from 60 to 90, the compressive strength has been marginally increased; however, increase of aspect ratio from 90 to 120 has not been found favorable and compressive strength has been slightly decreased. Although with the fibers of high aspect ratio the compressive strength has been increased, it is still slightly lesser than control (00-0). This may be explained by considering the fiber and concrete matrix interface. As the addition of fibers in the presence of coarse aggregates tends to create voids in the concrete and results improper packing of material in the concrete. The improper packing of material due to addition of fibers causes decrease in compressive strength. Though the volume fraction of fibers of different aspect ratio is same, the numbers of fibers are different due to variation in length and diameter. For example, the numbers of fibers of aspect ratio 45 due to longest length and higher diameter are lesser than the other fibers in the mix having the same volume fraction of fibers. Additionally, PVA fibers of aspect ratio 45 have been found to create less resistance in mixing and give better workability [28], while fiber of aspect ratio 120 has been found to create maximum resistance during mixing and gives least workability [28]. Based on this tendency, it may comprehend that PVA fibers of aspect ratio up to 90 are better in terms of compressive strength. The coarser type PVA fibers provide good workability and results in least problems of workability in the concrete in the presence of coarse aggregates and provides compressive strength comparable to concrete without fibers.

In HSDC, the target is to reduce the cement content and induce ductility without compromising the performance. To provide better packing without increasing the cement content and with the coarse aggregate, mineral admixture metakaolin has been utilized to increase the paste in the concrete. As shown in Figures 2 and 3, the compressive strength of concrete with 5% and 10% MK has been increased about 5% and 16.5% at age of 28 days as compared to control (00-0), respectively. The high surface area and fineness of MK are responsible for this behavior. MK, together with PVA fibers of aspect ratios 45, 60, and 90 up to 2% fiber volume fraction, has been found to be comparable with control (00-0). Only PVA fibers of aspect ratio 45 substantially increased the compressive strength in comparison with 5% MK concrete (00-5) as shown in Figure 2. Concrete with fiber having aspect ratio 120 (D1-5, D2-5) showed lesser strength as compared to concrete with PVA fibers of smaller aspect ratio. The reason for this trend is the fact that concrete mix has the relatively lesser workability with increase in fiber volume and aspect ratio. By keeping the water/binder ratio constant, concrete with a larger aspect ratio gives lesser workability [28] and tends to reduce the packing of material and hence gives lesser strength. MK through vibration or pumping demonstrated better workability similar to silica fume [29]. Therefore, the situation improves little bit with 10% MK in concrete mix (D1-10, D2-10) because of the increase of binder in the mix as shown in Figure 3. By comparing the results in Figures 2 and 3, from aspect ratio 60 to 90 with 5% and 10% MK, compressive strength of HSDC is consistent and compressive strength of HSDC with PVA fibers of aspect ratio 120 has been reduced.

**Figure 4:**
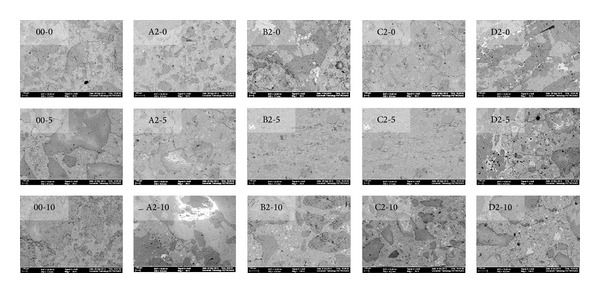
Microstructure of HSDC through field emission scanning electron microscope (FESEM).

The behavior of MK and PVA fibers may also be understood by images of microstructure of concrete through field emission scanning electron microscope (FESEM) as shown in Figure 4. The images of plain concrete with/without MK and with/without PVA fibers of different aspect ratio with 2% fiber volume fraction have been shown in Figure 4. The small black spots in the images are due to microvoids in the concrete. By comparing first column of the images which is without PVA fibers, it is clear that with MK microstructure has been refined and packing of material is much better with MK which is also reported by Poon et al. [12]. There are none to negligible black spots in concrete with 10% MK. After this, the second column, in which images of concrete with PVA fibers of aspect 45 have been presented, has more refined microstructure. From left to right, the images have been arranged in the order of increasing aspect ratio and it is quite clear that microstructure has suffered with the increase in aspect ratio. There are quite numbered black spots (voids) in concrete having PVA fibers of aspect ratio 120. Hence, the results of the microstructure of HSDC depicted in Figure 4 are coherent with the cube compressive strength.

**Figure 5:**
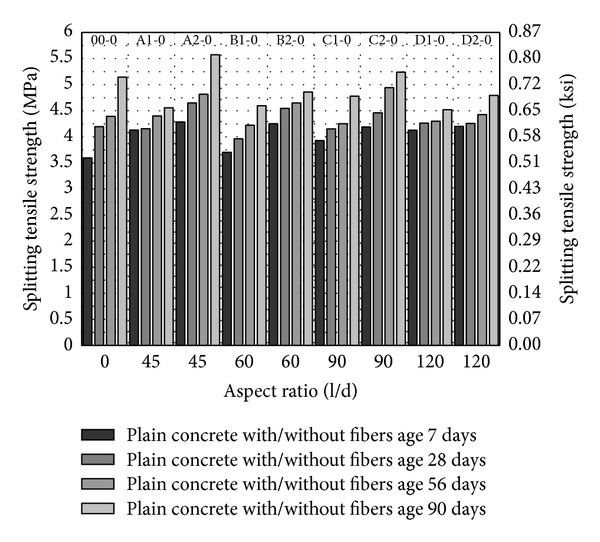
Splitting tensile strength of HSDC without metakaolin.

**Figure 6:**
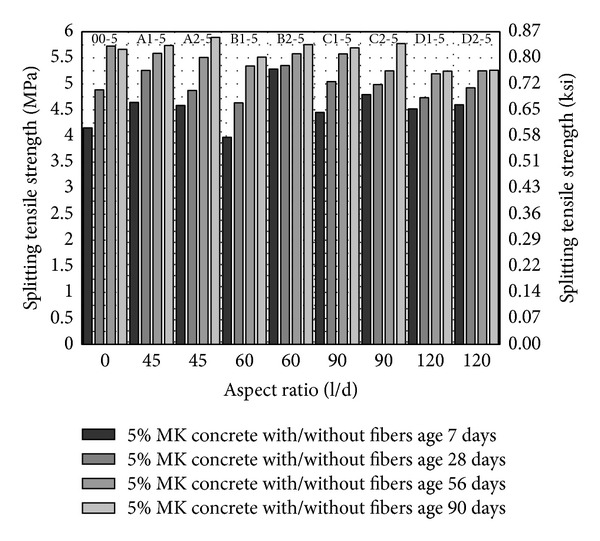
Splitting tensile strength of HSDC with 5% metakaolin.

**Figure 7:**
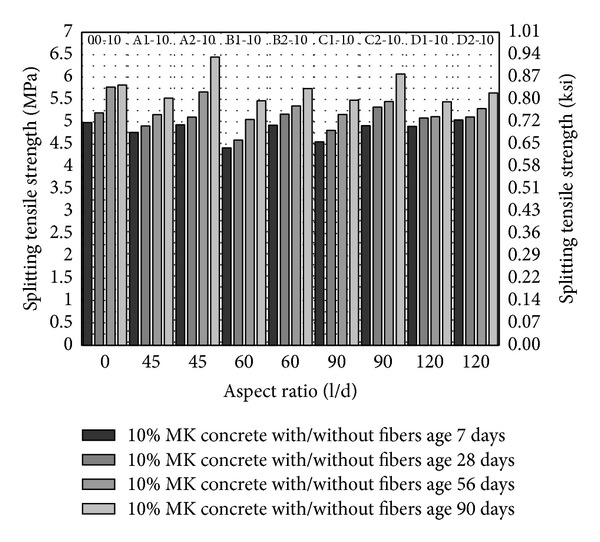
Splitting tensile strength of HSDC with 10% metakaolin.

From Figures 5, 6 and 7, splitting tensile strength has been plotted against fiber aspect ratio and the fiber volume fraction has been indicated as a series. Similar to compressive strength, splitting tensile strength increases with the age and with increase in volume fraction of fibers from 1 to 2%. In comparison with control (00-0) splitting tensile strength of concrete is increasing with the increase in the aspect ratio up to 90 with fiber volume fraction 1 to 2% as shown in Figure 5; however, the maximum splitting strength has been observed with aspect ratio 45 with 2% fiber volume fraction. It may infer that increase in aspect ratio and volume fraction of fibers gives higher splitting tensile strength.

Inclusion of 5 and 10% MK (00-5 and 00-10) caused 16.5% and 24% increase in splitting tensile strength at age of 28 days as compared to control (00-0) as shown in Figure 5 to Figure 7. This indicates that MK is providing dense matrix to concrete that increases the lateral confinement as well and results in higher splitting tensile strength. 5% MK together with PVA fibers has higher splitting tensile strength with PVA fiber having aspect ratio 45, 60, and 90 with 2% volume fraction. Even though the fiber showed slightly lesser or comparable strength as compared to 5% MK without fibers (00-5), the splitting tensile strengths are quite higher than control (00-0). Also, from aspect ratio 60 to 90, there is a trend of increase in splitting tensile strength with 5% MK which is similar to compressive strength. 10% MK gives peak splitting tensile strength with PVA fibers having aspect ratio 45 and 2% volume fraction as shown in Figure 7. In general all mixes with fiber showed higher strength with 10% MK as compared to control (00-0). Also, splitting tensile strength is more consistent and higher in early age with 10% MK and PVA fibers as compared to 5% MK and PVA fibers. With the increase in volume fraction and aspect ratio of fibers, 10% MK concrete demonstrated an increase in splitting tensile strength. From these results, it may be suggested that 10% MK has better response with the PVA fibers and has a more consistent and higher early strength.

**Figure 8:**
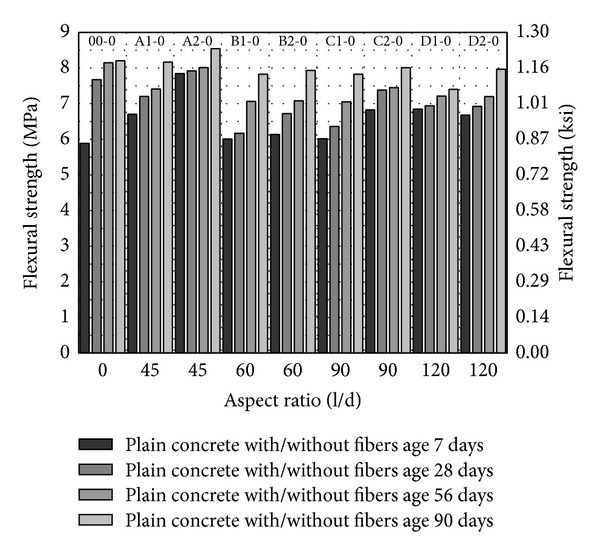
Flexural strength of HSDC without metakaolin.

**Figure 9:**
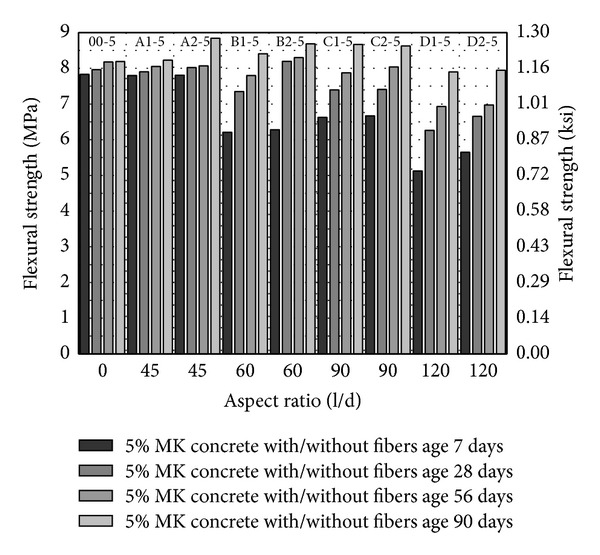
Flexural strength of HSDC with 5% metakaolin.

**Figure 10:**
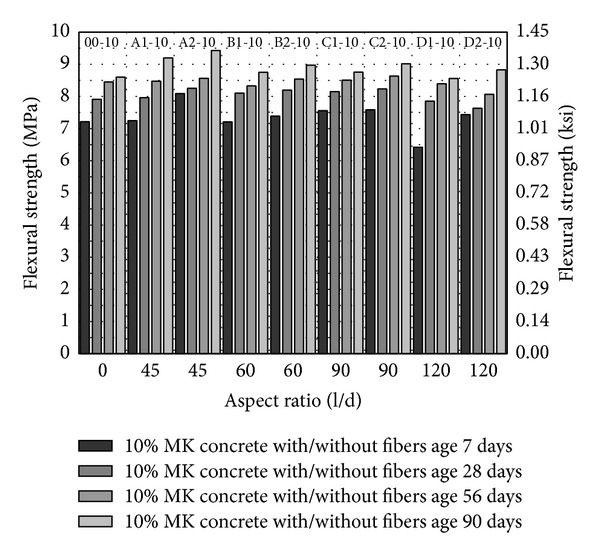
Flexural strength of HSDC with 10% metakaolin.

From Figures 8, 9, and 10, flexural strength has been plotted against fiber aspect ratio and the fiber volume fraction has been indicated as a series. Similar to compressive strength and splitting tensile strength, flexural strength increases with the age and with increase in volume fraction of fibers from 1 to 2%. In comparison with control (00-0), flexural strength of concrete with fibers has been increased about 3% with fiber volume fraction 2% having aspect ratio 45 (A2-0). With PVA fibers of other aspect ratio, either the flexural strength has been found to be competitive to control or lesser than control as shown in Figure 8. Also, increase in aspect ratio from 60 to 120 and volume fraction of fibers gives a marginal increase in flexural strength as shown in Figure 8.

With 5 and 10% MK (00-5 and 00-10), the seven-day flexural strength has been increased about 33% and 22% in relation to concrete without MK as shown in Figure 8 to Figure 10. This is very important for construction schedule view point as formwork and temporary supports would be required for lesser days in HSDC. On the later age, though flexural strength is increasing, the difference as compared to control (00-0) is marginal. With 5% MK, increase in aspect ratio 60 to 120 and volume fraction 1 to 2% of fibers decrease the flexural strength, while fiber of aspect ratio 45 has different attribute (length and diameter) and does not fit in this scheme. 10% MK together with fibers having aspect ratios 45, 60, and 90 with 2% volume fraction have higher flexural strength among all concrete mix studied as shown in Figure 8 to Figure 10. About 7.5% and 4.3% increase in flexural strength have been observed with fibers having aspect ratio 45, 2% volume fraction, and 10% MK (A2-10) as compared to control 00-0 and 00-10, respectively. In general, with 10% MK, increase in aspect ratio from 60 to 90 and volume fraction of 1 to 2% of fibers increases the flexural strength.

**Figure 11:**
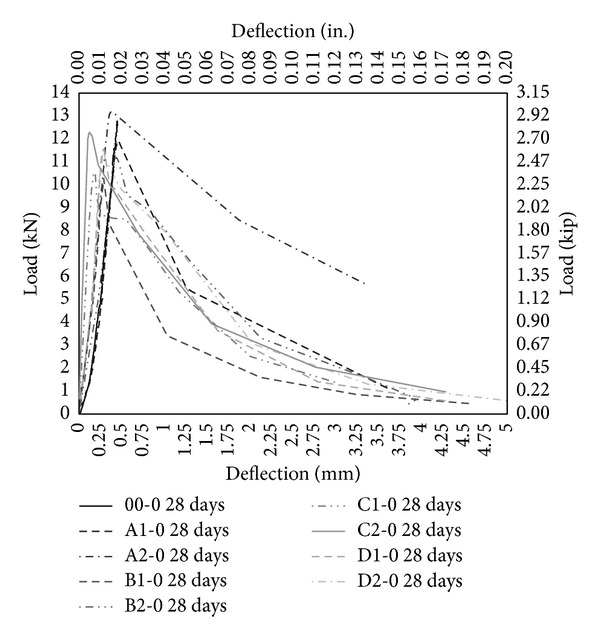
Load deflection under bending without metakaolin.

**Figure 12:**
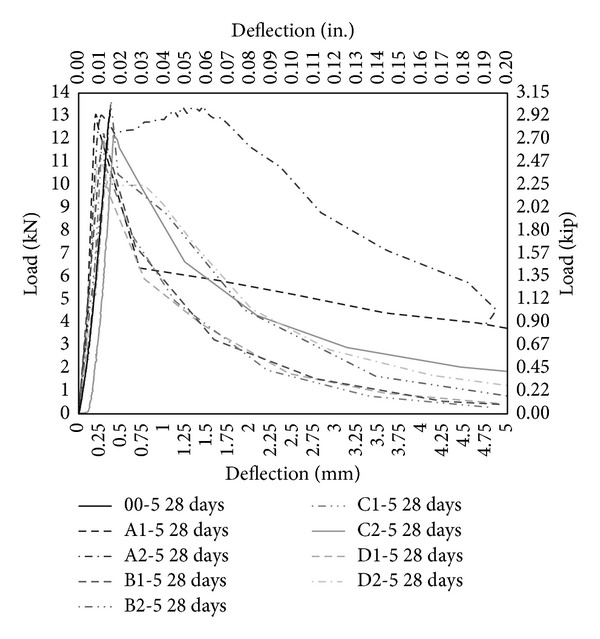
Load deflection under bending with 5% metakaolin.

**Figure 13:**
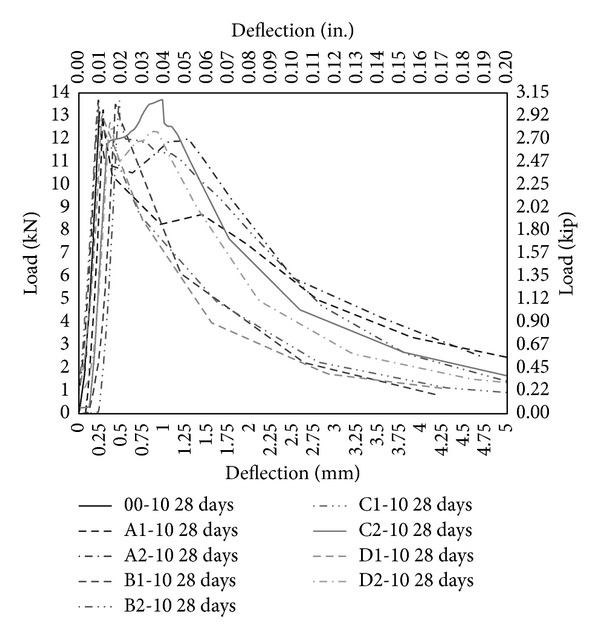
Load deflection under bending with 10% metakaolin.

The performance of PVA fibers has also been illustrated through load-deflection diagram under bending as shown in Figures 11, 12, and 13. PVA fibers not only give higher stiffness but also demonstrate the postpeak-load trend that is absent in case of control (00-0) and concrete with only MK (00-5, 00-10). Most of postpeak-load deflection of concrete with only PVA fibers prolongs to 4 mm as shown in Figure 11, but the load carrying capacity is lesser than control as shown in Figure 11. 5% MK together with PVA fibers further enhances the response under bending and most postpeak-load deflection reaches 5 mm and the load carrying capacity has been increased and now it is comparable to control (00-0). Moreover, with PVA fiber having aspect ratio 45 and 2% volume fraction (A2-5), there is a remarkable deflection hardening zone showing that fibers are pulling and stretching and sustaining the load up to reasonable deflection. 10% MK together with PVA fibers continues to improve the response under bending and gives the maximum load carrying capacity. Stiffness has been improved further on these concrete mixes and most postpeak-load deflections reached to 6 mm. Deflection hardening is further pronounced in almost all the mix with 10% MK. As reported in the literature that it is mandatory for a member to undergo large deflection before failure and it is the minimum requirement of flexural member and hence HSDC with the deflection hardening response satisfied the minimum requirement for structural applications [30]. Also, it has been reported that deflection hardening response is achieved with moderate fiber volume content (up to 2%) and it is used in beams with conventional reinforcement such as in seismic resisting structures [31]. Therefore, HSDC with 2% volume fraction and 10% MK is sustainable concrete having deflection hardening response. Though deflection hardening response is evident in HSDC, strain hardening or softening behavior is required to test in direct tension.

The postpeak-load behavior may further be described by comparing the flexural toughness of concrete. The area under a load-deflection curve is the flexural toughness for the beam tested in third-point bending. Toughness indices have been calculated as per ASTM C-1018 and ACI 544 as shown in Table 4. Toughness Index I for fiber-reinforced concrete reflects the improvement in flexural toughness over the plain concrete. In the concrete mix without MK, the toughness indices are maximum with fiber of aspect ratio 90 with 1 and 2% volume fraction (C1-0, C2-0). With 5% MK, the toughness indices are maximum with the fiber of aspect ratio 45 with 1 and 2% volume fraction (A1-5, A2-5) and their index values are higher than concrete without MK. Similarly, with 10% MK, the toughness indices are maximum with the fiber of aspect ratio 90 with 2% volume fraction (C2-10). Among all the mixes, the maximum toughness indices have been observed with 10% MK and 2% volume fraction as shown in Table 4. The maximum value of toughness index *I*_30_ is 15.81 in HSDC which is slightly lesser than the toughness index reported with steel fiber reinforced high strength concrete [32].

## 4. Conclusions and Recommendations

1. All the mixes of HSDC considered in this study have common trend, that is, all strengths have been increased with the age and with increase in volume fraction of fibers from 1 to 2%.
2. The highest cube compressive strength has been observed with 2% volume fraction of fibers having aspect ratio 45 and 5–10% MK (A2-5, A2-10).
3. PVA fibers of aspect ratio up to 90 are better in terms of compressive strength. The coarser type PVA fibers provide good workability and results in least problems of workability in the concrete in the presence of coarse aggregates and hence provides compressive strength comparable to concrete without fibers.
4. The compressive strength of concrete with 5% and 10% MK has been increased about 5% and 16.5% at age of 28 days as compared to control (00-0), respectively.
5. The microstructure of concrete with MK has been refined and packing of material is much better with MK which is also reported by Poon et al. [12]. There is none to negligible voids in concrete with 10% MK.
6. Concrete with PVA fibers of aspect 45 has also refined microstructure; however, the microstructure has suffered with the increase in aspect ratio of fibers.
7. In comparison with control (00-0) splitting tensile strength of concrete is increasing with the increase in the aspect ratio up to 90 with fiber volume fraction 1 to 2%. Based on this trend, it may infer that increase in aspect ratio and volume fraction of fibers gives higher splitting tensile strength.
8. Inclusion of 5 and 10% MK (00-5 and 00-10) caused 16.5% and 24% increase in splitting tensile strength at age of 28 days as compared to control (00-0).
9. 5% MK together with PVA fibers has higher splitting tensile strength with PVA fiber having aspect ratio 45, 60, and 90 with 2% volume fraction. Even though the fiber showed slightly lesser or comparable strength as compared to 5% MK without fibers (00-5), the splitting tensile strengths are quite higher than control (00-0).
10. 10% MK gives peak splitting tensile strength with PVA fibers having aspect ratio 45 and 2% volume. All mixes with fiber showed higher strength with 10% MK as compared to control (00-0). Also, splitting tensile strength is more consistent and higher in early age with 10% MK and PVA fibers as compared to 5% MK and PVA fibers. With the increase in volume fraction and aspect ratio of fibers, 10% MK concrete demonstrated an increase in splitting tensile strength.
11. In comparison with control (00-0), flexural strength of concrete with PVA fibers alone has been increased about 3% with fiber volume fraction of 2% having aspect ratio 45 (A2-0). With PVA fibers of other aspect ratio, the flexural strength has been found to be either competitive to control or lesser than control.
12. With 5 and 10% MK (00-5 and 00-10), the seven-day flexural strength has been increased about 33% and 22% in relation to concrete without MK. This is very important for construction schedule view point as formwork and temporary supports would be required for lesser days in HSDC.
13. 10% MK together with fibers having aspect ratio 45, 60 and 90 with 2% volume fraction have higher flexural strength among all concrete mix. About 7.5% and 4.3% increase in flexural strength have been observed with fibers having aspect ratio 45, 2% volume fraction, and 10% MK (A2-10) as compared to control 00-0 and 00-10, respectively.
14. PVA fibers not only give higher stiffness but also showed the postpeak-load trend that is absent in case of control (00-0) and concrete with only MK (00-5, 00-10).
15. Most of postpeak-load deflections of concrete with only PVA fibers prolong to 4 mm but the load carrying capacity is lesser than control; however, 5% MK together with PVA fibers not only increased the postpeak-load deflection but also increased the load carrying capacity.
16. 10% MK together with PVA fibers continues to improve the response under bending and gives the maximum load carrying capacity. Stiffness has been improved further on these concrete mixes and most postpeak-load deflection reached to 6 mm. Deflection hardening is further pronounced in almost all the mix with 10% MK.
17. HSDC with 2% volume fraction and 10% MK is sustainable concrete having deflection hardening response. Toughness Index I for fiber-reinforced concrete reflects the improvement in flexural toughness over the plain concrete.
18. Among all the mix, the maximum toughness indices have been observed with 10% MK and 2% volume fraction. The maximum value of toughness index *I*_30_ is 15.81 in HSDC which is slightly lesser than the toughness index reported with steel fiber reinforced high strength concrete [32].

## Figures and Tables

**Table 1:** Chemical and physical properties of OPC and mineral admixtures (%) [29].

	OPC	FA	GGBS	SF	MK	RHA
Specific gravity	3.05	2.2–2.8	2.79	2.6–3.8	2.5	2.11

SiO_2_, %	20.44	35–60	34.4	91.4	53.87	88.32
Al_2_O_3_, %	2.84	10–30	9.0	0.09	38.57	0.46

Fe_2_O_3_, %	4.64	4–20	2.58	0.04	1.4	0.67
CaO, %	67.73	1–35	44.8	0.93	0.04	0.67
MgO, %	1.43	1.98	4.43	0.78	0.96	0.44
SO_3_, %	2.20	0.35	2.26	0.01	—	—
Na_2_O, %	0.02	0.48	0.62	0.39	0.04	0.12
K_2_O, %	0.26	0.4	0.5	2.41	2.68	2.91
MnO, %	0.16	—	—	0.05	0.01	—
TiO_2_, %	0.17	—	—	0.0	0.95	—

Particle size, *µ*m	10–40	≤45	—	0.1	0.5–20	11.5–31.3

Specific surface (m^2^/g)	1.75	5–9	0.4–0.599	16.455	12.174	30.4–27.4

FA: fly ash, GGBS: ground granulated blast furnace slag, SF: silica fume, RHA: rice husk ash, MK: metakaolin.

**Table 2:** Properties of reinforcing fibers for cementitious composites [16, 17, 33].

Fiber type	Tensile strength (MPa)	Young's modulus (GPa)	Fiber elongation (%)	Specific gravity	Remarks
PVA fiber	880~1600	25~40	6~10	1.3	—
Polypropylene	500~700	6~7	20	0.91	—
Nylon fiber	750~900	3.4~4.9	13~25	1.10	—
Steel fiber	1200	200	3~4	7.85	Heavy, rusts
Glass fiber	2200	80	0~4	2.78	Weak in alkali
Asbestos	620	160	0.6	2.55	Health risky
Kevlar	3600	65	4.1	1.45	Costly, specialized use
Basalt fiber	992	7.6	2.56	2.6	—

**Table 3:** Nomenclature and detail of mix proportion of HSDC.

MIX	MK	OPC	Fine aggregates	Superplasticizer	Coarse aggregates		PVA fiber		
					<10 mm	10–20 mm	weight	*V*_*f*_	Aspect ratio
	kg/m^3^	kg/m^3^	kg/m^3^	%	kg/m^3^	kg/m^3^	kg/m^3^	%	length/dia
00-0	0	450	670	0.25	600	500	0	0	—
A1-0	0	450	670	1	600	500	13	1	45
A2-0	0	450	670	1.25	600	500	26	2	
B1-0	0	450	670	1	600	500	13	1	60
B2-0	0	450	670	1.25	600	500	26	2	
C1-0	0	450	670	1	600	500	13	1	90
C2-0	0	450	670	1.25	600	500	26	2	
D1-0	0	450	670	1	600	500	13	1	120
D2-0	0	450	670	1.25	600	500	26	2	

00-5	22.5	427.5	670	0.5	600	500	0	0	—
A1-5	22.5	427.5	670	1	600	500	13	1	45
A2-5	22.5	427.5	670	1.25	600	500	26	2	
B1-5	22.5	427.5	670	1	600	500	13	1	60
B2-5	22.5	427.5	670	1.25	600	500	26	2	
C1-5	22.5	427.5	670	1	600	500	13	1	90
C2-5	22.5	427.5	670	1.25	600	500	26	2	
D1-5	22.5	427.5	670	1	600	500	13	1	120
D2-5	22.5	427.5	670	1.25	600	500	26	2	

00-10	45	405	670	0.75	600	500	0	0	—
A1-10	45	405	670	1	600	500	13	1	45
A2-10	45	405	670	1.25	600	500	26	2	
B1-10	45	405	670	1	600	500	13	1	60
B2-10	45	405	670	1.25	600	500	26	2	
C1-10	45	405	670	1	600	500	13	1	90
C2-10	45	405	670	1.25	600	500	26	2	
D1-10	45	405	670	1	600	500	13	1	120
D2-10	45	405	670	1.25	600	500	26	2	

**Table 4:** Toughness indices at various volume fraction and aspect ratio of fibers.

Mix	Aspect ratio	Fiber lengt	Fiber diameter	Toughness indices as per ASTM C-1018			Toughness index as per ACI 544
	*l*/*d *	h mm	mm	*I*_5_	*I*_10_	*I*_30_	*I*
00-0	—	—	—	1.0	1.0	1.0	1.0
A1-0	45	30	0.667	1.09	1.7	2.31	2.7
B1-0	60	12	0.2	2.1	3.35	4.38	4.35
C1-0	90	18	0.2	2.0	4.81	8.15	7.91
D1-0	120	24	0.2	2.45	4.55	6.69	5.55
A2-0	45	30	0.667	3.88	5.6	6.38	7.38
B2-0	60	12	0.2	2.98	4.57	6.04	5.57
C2-0	90	18	0.2	3.45	5.64	9.93	10.93
D2-0	120	24	0.2	2.95	5.04	7.34	6.41

00-5	—	—	—	1.0	1.0	1.0	1.0
A1-5	45	30	0.667	3.5	7.39	14.77	11.09
B1-5	60	12	0.2	2.77	3.88	5.89	5.53
C1-5	90	18	0.2	3.4	4.98	6.64	6.1
D1-5	120	24	0.2	3.44	5.13	6.85	6.13
A2-5	45	30	0.667	3.37	6.45	13.36	7.45
B2-5	60	12	0.2	2.89	4.27	5.48	4.42
C2-5	90	18	0.2	1.49	2.1	2.97	2.63
D2-5	120	24	0.2	3.30	6.35	11.5	9.56

00-10	—	—	—	1.0	1.0	1.0	1.0
A1-10	45	30	0.667	2.48	5.68	10.6	6.67
B1-10	60	12	0.2	3.17	4.07	5.03	4.62
C1-10	90	18	0.2	1.77	3.8	5.96	5.8
D1-10	120	24	0.2	3.48	4.08	6.41	6.17
A2-10	45	30	0.667	2.36	4.73	9.43	6.8
B2-10	60	12	0.2	5.51	9.06	12.57	8.02
C2-10	90	18	0.2	5.82	9.11	15.81	10.11
D2-10	120	24	0.2	3.61	5.23	8.03	6.23
